# Current Approaches to Worsening Heart Failure: Pathophysiological and Molecular Insights

**DOI:** 10.3390/ijms25031574

**Published:** 2024-01-26

**Authors:** Andrea D’Amato, Silvia Prosperi, Paolo Severino, Vincenzo Myftari, Aurora Labbro Francia, Claudia Cestiè, Nicola Pierucci, Stefanie Marek-Iannucci, Marco Valerio Mariani, Rosanna Germanò, Francesca Fanisio, Carlo Lavalle, Viviana Maestrini, Roberto Badagliacca, Massimo Mancone, Francesco Fedele, Carmine Dario Vizza

**Affiliations:** 1 Department of Clinical, Internal, Anesthesiology and Cardiovascular Sciences, Sapienza University of Rome, 00161 Rome, Italy; damatoandrea92@gmail.com (A.D.); silviapro@outlook.it (S.P.); vincenzo.myftari@gmail.com (V.M.); auro1298@gmail.com (A.L.F.); claudia.cestie@gmail.com (C.C.); npierucci@gmail.com (N.P.); stefanie.marekiannucci@gmail.com (S.M.-I.); marcoval.mariani@gmail.com (M.V.M.); rosanna.germano@gmail.com (R.G.); carlo.lavalle@uniroma1.it (C.L.); viviana.maestrini@uniroma1.it (V.M.); roberto.badagliacca@uniroma1.it (R.B.); massimo.mancone@uniroma1.it (M.M.); dario.vizza@uniroma1.it (C.D.V.); 2 Division of Cardiology, Policlinico Casilino, 00169 Rome, Italy; fanisio.francesca@gmail.com; 3 San Raffaele Cassino, 03043 Cassino, Italy; francesco.fedele@uniroma1.it

**Keywords:** worsening heart failure, levosimendan, vericiguat, SGLT2i, omecamtiv mecarbil, cardiac contractility modulation

## Abstract

Worsening heart failure (WHF) is a severe and dynamic condition characterized by significant clinical and hemodynamic deterioration. It is characterized by worsening HF signs, symptoms and biomarkers, despite the achievement of an optimized medical therapy. It remains a significant challenge in cardiology, as it evolves into advanced and end-stage HF. The hyperactivation of the neurohormonal, adrenergic and renin-angiotensin-aldosterone system are well known pathophysiological pathways involved in HF. Several drugs have been developed to inhibit the latter, resulting in an improvement in life expectancy. Nevertheless, patients are exposed to a residual risk of adverse events, and the exploration of new molecular pathways and therapeutic targets is required. This review explores the current landscape of WHF, highlighting the complexities and factors contributing to this critical condition. Most recent medical advances have introduced cutting-edge pharmacological agents, such as guanylate cyclase stimulators and myosin activators. Regarding device-based therapies, invasive pulmonary pressure measurement and cardiac contractility modulation have emerged as promising tools to increase the quality of life and reduce hospitalizations due to HF exacerbations. Recent innovations in terms of WHF management emphasize the need for a multifaceted and patient-centric approach to address the complex HF syndrome.

## 1. Introduction

**Figure 1:**
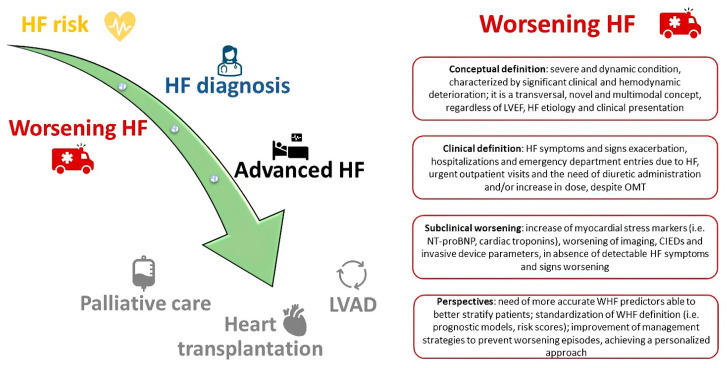
The clinical behaviour of HF syndrome and the main features of WHF. In the left side of the figure, HF is represented as a progressive disease. In the right side of the figure, the main features of WHF are represented. A distinction between the conceptual and the clinical definition is made. Subclinical worsening is often underdiagnosed in clinical practice and its identification may prevent adverse outcomes. The improvement of WHF management should involve the creation of a personalized, patient-tailored approach. HF: heart failure; WHF: worsening heart failure; LVAD: left ventricular assist device; LVEF: left ventricular ejection fraction; OMT: optimized medical therapy; NT proBNP: N-terminal pro B-type natriuretic peptide; CIEDs: cardiac implantable electronic devices; WHF: worsening heart failure.

Heart failure (HF) is a syndrome defined by structural abnormalities and/or functional heart dysfunction, leading to inadequate cardiac output and/or increased intraventricular filling pressure [1,2]. HF affects millions of patients worldwide, with an increasing incidence, representing one of the most common causes of hospitalization and death [1,2]. HF is a multisystemic disease, initially affecting the heart and progressively involving other organs, such as lungs and kidneys, eventually leading to multiorgan dysfunction. HF may be considered a progressive disease, characterized by worsening episodes despite optimized medical therapy (OMT). The episodes of exacerbation are increasingly frequent. They are associated with the worsening of patient’s clinical and functional status, leading to death or the need for heart transplantation and/or left ventricular assist device implantation. Therefore, the term of “worsening HF (WHF)” has been introduced in clinical practice and the scientific literature [3,4]. WHF is a severe and dynamic condition characterized by significant clinical deterioration and a worsening prognosis. The definition of WHF encompasses recurrent hospitalizations and emergency department entries due to HF, urgent outpatient visits and the need for intravenous diuretic administration [3,4]. There are other conditions not yet included in the WHF definition which are suggestive of disease progression, such as the increased need for oral diuretics. The current definition of WHF does not include subclinical worsening, which often occurs in clinical practice. This is characterized by an increase in myocardial stress markers, such as N-terminal pro B-type natriuretic peptide (NT-proBNP) and cardiac troponins, preceding the occurrence of new clinical symptoms, and it is often undertreated by cardiologists [3]. WHF is a transversal, novel and multimodal concept. It involves patients regardless of left ventricular ejection fraction (LVEF), HF aetiology and previous presentation (Figure 1).

For decades, hospitalization represented the main outcome and focus regarding HF [5]. However, hospitalization is the result of a progressive worsening which occurs outside of the hospital. Outpatient management is crucial, including urgent ambulatory visits for the management of diuretics and OMT, to halt or reduce the worsening of the disease [5]. Ambrosy et al. [6] identified that WHF was more common than hospitalization itself and was associated with a high mortality burden. Therefore, meticulous management of worsening HF and prevention should have priority over the other aspects of HF [5,6,7]. The abovementioned concept further strengthens the limitations of the actual classification and prognostic models used for HF [8,9,10,11].

The European Society of Cardiology (ESC) and the American College of Cardiology/American Heart Association (ACC/AHA) [1,2] published guidelines for the diagnosis and treatment of acute and chronic HF, proposing four classes of drugs for HF with reduced ejection fraction (HFrEF): beta blockers (BB), mineralocorticoid receptor antagonists (MRAs), angiotensin converting enzyme inhibitors/angiotensin receptor-neprilysin inhibitors (ACEi/ARNIs) and sodium-glucose cotransporter-2 inhibitors (SGLT2i), in order to reduce mortality and hospitalization risk. Recent evidence [12,13] and the recent update of the HF guidelines [14] focus on the importance of an upfront and quick up-titration of those agents, to reach their maximum benefits. However, despite OMT, there is a non-neglectable part of the population exposed to the residual risk of unpredictable hospitalization and mortality [15]. Therefore, new therapeutic approaches, representing adjunctive therapeutic tools against HF, will emerge in the near future.

The aim of this review is to revise the pathophysiological basis of the current and coming options for the management of patients with WHF.

## 2. Repetitive Levosimendan Infusion in Worsening Heart Failure: The Achievement of Haemodynamic Stabilization

The inodilator Levosimendan is a calcium sensitizer exhibiting pleiotropic effects. Its mechanism of action is related to a metabolite which is subsequently acetylated in the liver into an active metabolite with a long half-life named OR-1896 (the negative enantiomer of N-[4-[4-(1,4,5,6-tetrahydro-4-methyl-6-oxo-3-pyridazinyl) phenyl] acetamide). OR-1896 is reported to have a plasma protein binding capacity of 40% and a half-life of 75–80 h, thus explaining the persistence of cardiovascular effects for 7–9 days following discontinuation of the drug’s administration. At the molecular level, the inotropic action of Levosimendan is performed by sensitizing cardiac troponin C (cTnC) to calcium (Ca^2+^) binding, and by determining the conformation that cTnC usually assumes after Ca^2+^ binding [16]. cTnC activity turns out to be crucial in the kinetics of the systolic phase of the cardiac cycle and relaxation in the diastolic phase [17]. The calcium sensitizer enhances the contractility of cTnC exclusively in the Ca^2+^ activated conformation, thereby improving systolic function without affecting diastolic relaxation or augmenting the risk of adverse effects related to the increased Ca^2+^ concentration [16]. Both the drug itself and OR-1896 act at the vascular level by causing hyperpolarization of smooth muscle cells through activation of adenosine triphosphate -dependent potassium (K-ATP) channels. This results in vasodilation of pulmonary, coronary and peripheral arteries. An endothelial component also seems to be involved in the vasodilation, and an interaction between K-ATP and nitric oxide (NO) formation has been identified [17]. Levosimendan and its metabolite are also found to be selective inhibitors of phosphodiesterase isoenzyme 3 (PDE 3), potentially resulting in an increase in intracellular Ca^2+^ concentration. In experimental models, it has been shown that a part of the Levosimendan action its exerted via a cyclic adenosine monophosphate (cAMP)-dependent mechanism by inhibiting PDE 3 [16].

**Figure 2:**
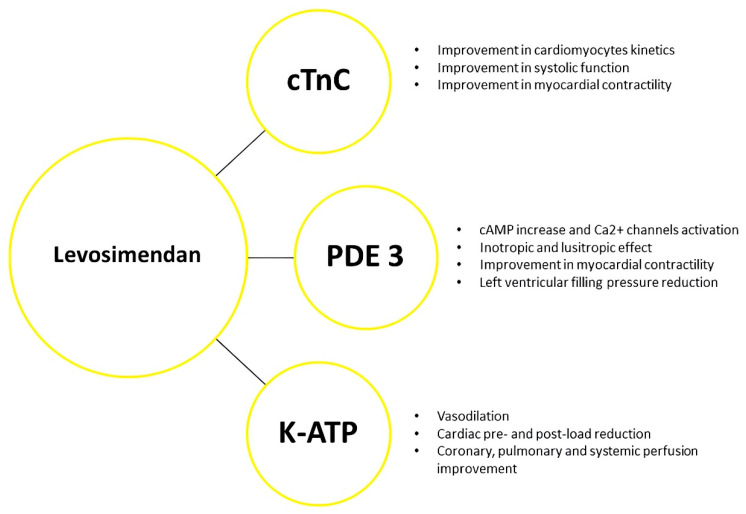
Levosimendan and main molecular targets. Levosimendan sensitizes cTnC to Ca^2+^ binding, inhibits PDE 3 and activates K-ATP channels. The cumulative effect consists of improvement in myocardial contractility and reduction in left ventricular filling pressure and coronary, pulmonary and systemic perfusion, without increasing the myocardial oxygen consumption. cTnC: cardiac troponin C; PDE 3: phosphodiesterase 3; K-ATP: ATP-dependent K^+^ channels; cAMP: cyclic adenosine monophosphate.

The increase in Ca^2+^ concentration is strictly dependent on the phosphodiesterase isoforms located in the cells and their localization within the cell, based on which, selective inhibition of PDE 3 may have variable effects in increasing Ca^2+^ concentration [17]. A moderate increase in intracellular Ca^2+^ concentration may have an inotropic effect [16] (Figure 2). Moreover, Levosimendan exerts beneficial effects on several other organs, such as inducing an improvement in renal function [18] and peripheral organ perfusion, which made it a key drug in the management of critical ill patients [19].

The potential benefit of intermittent administration of Levosimendan in WHF has been studied. The rationale for intermittent administration can be attributed to the pharmacological and hemodynamic properties of the calcium-sensitizer, but especially to the presence of the active metabolite OR-1896, which exhibits a peak plasma concentration approximately 80 h after administration, thus explaining the need for weekly administration [20]. Several studies were conducted to assess the efficacy and safety of Levosimendan administration in WHF, reporting controversial results. The LevoRep trial [20] was a multicentre, randomized, prospective, double-blind trial whose primary endpoint was a composite of at least a 20% improvement in the 6-min walk test (6MWT) and at least a 15% improvement in the Kansas City Cardiomyopathy Questionnaire (KCCQ-12) following repetitive infusions of Levosimendan in an acute HF outpatient setting. The patients enrolled had to have a diagnosis of heart failure of New York Heart Association (NYHA) class III-IV for at least three months, OMT, a LVEF ≤ 35% and a distance of < 350 m in the 6MWT. The study included 120 patients, which were randomized to either receive Levosimendan or placebo. Of the patients, 63 were treated with Levosimendan, and 57 with placebo. Great emphasis was put on adverse events. Relating to the safety of the proposed treatment, it was shown that the frequency of side effects was comparable in the two study groups in terms of tachycardia, non-sustained ventricular tachycardia and new-onset atrial fibrillation. Median drop in systolic blood pressure was reported to be more significant in the Levosimendan group compared to the placebo group. Altenberger et al. [20] reported that the intermittent outpatient treatment did not significantly improve patients’ functional capacity and quality of life when compared to placebo administration. The LION-HEART trial [21] was a multicentre, randomized, double-blind trial which included 69 patients who underwent randomization at a 2:1 ratio to be treated either with Levosimendan or with placebo. Hence, 48 were allocated to Levosimendan, and 21 to placebo. The main inclusion criteria were age > 18 years, LVEF < 35% in the previous 6 months and clinical diagnosis of advanced chronic HF. The primary outcome of the study was the assessment of serum NT-proBNP concentration during 12 weeks of treatment. Secondary outcomes included drug safety, patient-reported effects and clinical events. Levosimendan or placebo was administered every 2 weeks for 12 weeks by means of a 6-h intravenous infusion. Comín-Colet et al. [21] demonstrated that the intermittent administration of Levosimendan was effective in decreasing the serum concentration of NT-proBNP and was associated with improvement in clinical symptoms in comparison to the placebo group. The LAICA study [22] was a multicentre trial aimed at evaluating the efficacy and safety of intermittent administration of Levosimendan as a continuous, 24-h, intravenous infusion in patients with advanced HF. The infusion was administered once a month for 12 months. The primary endpoint was the incidence of rehospitalization for acute decompensated HF or clinical deterioration of the underlying HF. The secondary endpoints were time from randomization to first hospitalization due to acute decompensated HF and/or death; cumulative incidence of hospitalization due to decompensated HF, death or both; changes in NYHA class throughout follow-ups; NT-proBNP changes before and after treatment; quality of life (QoL) assessment throughout the follow-ups. The study included 213 patients, which were randomized in a 3:1 fashion to either receive Levosimendan or placebo. In the study, 163 patients received Levosimendan, and 50 patients received placebo. Eligible patients had advanced HF: severe left ventricular dysfunction of any aetiology, with at least one episode of acute decompensation which required hospital admission within the past 6 months. García-González et al. [22] demonstrated the beneficial impact of repetitive Levosimendan infusion in reducing the HF hospitalization rate and HF worsening in patients with advanced HF, compared with placebo. The LeoDOR trial [23] was a multicentre trial with the aim of evaluating the efficacy and safety of intermittent intravenous Levosimendan therapy, administered in addition to standard therapy, for 12 weeks, either as a 6-h continuous infusion or as a 24-h continuous infusion. The study included 264 patients, which were randomized in a 2:1 fashion to either receive Levosimendan or placebo. In the study, 88 patients were treated with a 6-h Levosimendan infusion, 88 patients were treated with a 24-h Levosimendan infusion, and 88 patients were treated with placebo. The primary endpoint was a composite of time to death, urgent heart transplantation or VAD implantation; time to a non-fatal HF event requiring intravenous vasoactive therapy; and time-averaged change in NT-proBNP from baseline to 14 weeks. The trial found that the repetitive administration of Levosimendan in patients with a recent HF hospitalization, did not result in post-hospitalization clinical stability. 

Recently, Masarone et al. reported the role of Levosimendan as a “bridge to optimization” therapy, by facilitating the introduction of HF disease modifying drugs in patients with advanced HF who were previously intolerant [24].

In summary, the repetitive administration of Levosimendan and its role to achieve the hemodynamic stabilization in patients with worsening HF remain controversial, and further studies are needed, especially considering the limited number of participants in the above-mentioned studies, taking into account the wide spectrum of advanced HF patient profiles and the complexity of their management.

## 3. Innovative Pharmacological Targets in Worsening Heart Failure

**Figure 3:**
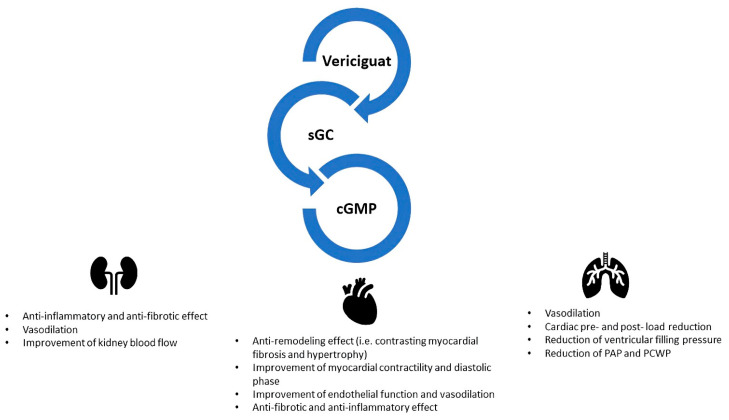
Vericiguat and main pathophysiological effects. Vericiguat has pleiotropic effects. It is an sGC enhancer which acts regardless of NO presence. It induces an increase in cGMP activating several molecular pathways. It acts by improving myocardial energetics, exploiting an anti-remodelling, anti-inflammatory and anti-fibrotic effect. It reduces pre- and post-load left ventricular filling pressures, improving also kidney blood flow. sGC: soluble guanylate cyclase; cGMP: cyclic guanosine monophosphate; PAP: pulmonary arterial pressure; PCWP: post-capillary wedge pressure.

Vericiguat is a cyclic guanosine monophosphate (cGMP) pathway enhancer, which acts by directly stimulating soluble guanylate cyclase (sGC). Under normal conditions, the vascular endothelium generates NO, which stimulates the sGC-mediated production of cGMP [25]. The endocardial endothelium is responsive to NO and regulates the contractility and diastolic function by increasing cGMP [25]. cGMP leads to the activation of protein kinase G (PKG), which promotes downstream pathways leading to vasodilation, reduction in inflammation and fibrosis, inhibition of hypertrophy and reduction in cardiac remodelling [16]. Vericiguat’s relevance in the treatment of HF can be explained by the fact that NO dependent intracellular cGMP generation is hindered, since ongoing inflammation, oxidative stress and endothelial dysfunction reduce NO levels [26] (Figure 3). 

Therefore, contrary to other drugs used in the HF setting which aim to inhibit detrimental pathways, Vericiguat stimulates the protective NO-sGC-GMPc pathway. Results on Vericiguat were brought to light in 2020 by the VICTORIA trial [27], which included 5050 patients with chronic HF, a LVEF <45% and an episode of recent worsening HF. Results showed that, among patients with high-risk HF, the incidence of the primary endpoint, a composite of death from cardiovascular causes or first hospitalization for HF, was lower in those who received Vericiguat compared to those who received placebo [27]. More specifically, Vericiguat was shown to reduce the primary outcome risk by 10% in a population of patients which had been hospitalized for HF in the previous 6 months or which had received outpatient intravenous diuretics in the previous three months, despite high background guidelines-directed medical therapy (GDMT) [27]. Vericiguat was also proven to be safe and well tolerated, with no significant effects on kidney function and electrolytes, showing minimal effects on blood pressure upon treatment initiation, and, overall, few side effects compared to placebo [24,25,26,27]. Based on the VICTORIA trial [27] results, Vericiguat has been approved for the treatment of symptomatic chronic HF with a LVEF<45%, following a worsening HF event, with the objective of reducing cardiovascular death and HF hospitalization risk. The Vericiguat effect depends on the range of NT-proBNP, showing worse effects in case of very high values, and, on LVEF, showing less efficacy when it is very low [27]. It has been introduced as a Class IIb recommendation in 2021 ESC HF guidelines, to be used in patients with worsening HF despite optimized GDMT [1,27]. Currently, a trial, named the VICTOR trial, is ongoing to extend the indication of Vericiguat in patients with chronic HF, regardless of worsening episodes.

Many patients with worsening HF are at high risk of experiencing adverse clinical events leading to hospitalization, despite optimized GDMT. This is of relevance since each acute HF hospitalization tends to further deteriorate the patient’s clinical status and myocardial function, representing a relevant marker of disease progression. Hence, considering the high mortality and hospitalization rate of patients with WHF, and, considering the persistently high risk of adverse events despite GDMT, Greene et al. [28] suggest that the adjunct use of Vericiguat, added to standard quadruple GDMT, is to be considered. In clinical practice, patients currently often do not achieve up-titration to the target dosage of GDMT. However, the benefits of Vericiguat on mortality or HF hospitalization seem to be consistent independent of GDMT use and dose [28]. It should be noted that about 40% of patients in the VICTORIA trial [27] were classified as having NYHA class III and only 15% received a concomitant therapy with an ARNI. These findings seem to highlight the role of Vericiguat in a subset of fragile and highly symptomatic patients. Moreover, as it has been largely demonstrated that the benefits of the current four pillars of GDMT are additive, this applies to Vericiguat as well. Hence, the authors imply that initiation of Vericiguat may be evaluated either while reaching GDMT target doses or simultaneously with GDMT up-titration [28].

Senni et al. [29] identified WHF patients in the VICTORIA trial who may benefit more from Vericiguat administration according to NT proBNP value at baseline. Dividing the population into four NT proBNP quartiles (Q1–Q4), a reduction in the composite endpoint of cardiovascular death or HF first hospitalization was observed for Q1–Q3 compared to placebo (the NT proBNP values for each quartile are Q1, ≤1556 pg/mL, Q2, >1556–2816 pg/mL and Q3, >2816–5314 pg/mL), while Vericiguat seems to perform worse in patients of Q4 (NT proBNP values >5314 pg/mL) compared to placebo. These patients, who were advanced, with several comorbidities, and clinically unstable did not benefit from Vericiguat administration [29].

Omecamtiv Mecarbil (OM) is a cardiac myosin activator, a small molecule that enhances cardiac contractility without modifying intracellular Ca^2+^ concentration or myocardial oxygen consumption. Its mechanism of action is based on the binding to myosin [30]. The contraction of cardiac sarcomeres is based on the combination of three elements: ATP hydrolysis, the binding of myosin heads to actin filaments and the swing of the lever arm. The power stroke produces the shortening of sarcomeres based on various states: (i) Pi release; (ii) intermediate phase; (iii) strong-ADP; (iv) rigor state; (v) post-rigor state, consisting of the separation between actin and myosin and the subsequent ATP binding; (vi) pre-power stroke state based on ATP hydrolysis forming ADP + Pi. The latter is reversible, becoming later irreversible when the active site opens to release the Pi. OM acts first by shifting the balance from the recovery status to ADP + P status, thus recruiting more myosin heads ready to bind to the actin filament and augmenting the Pi release, without affecting the ADP release [30]. The binding site of OM was identified in the post rigor state; however, in a study based on bovine models, a binding site was identified in the pre-power stroke state. Given its electronic configuration, this allosteric site allows an unambiguous bond between myosin and OM. The binding pocket is available only when the lever arm is primed. Fundamentally, OM augments the myosin heads primed to bind to the actin filament, thus producing an increased contraction force soon after the binding between Ca^2+^ and troponin-tropomyosin complex [30,31,32]. Moreover, OM is responsible for the slowing of the non-productive turnover of ATP, thus reducing the use of ATP not associated with mechanical work [30].

**Figure 4:**
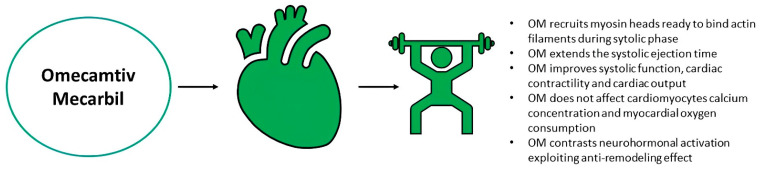
Omecamtiv Mecarbil and main pathophysiological effects. OM is an oral inotropic agent which stimulates myosin heads’ recruitment ready to bind actin filaments. It improves systolic function, cardiac contractility and systolic ejection time, without increasing myocardial oxygen consumption. OM: omecamtiv mecarbil.

In HFrEF, ventricular remodelling plays a key pathophysiological role. Within this patient population, the ventricular remodelling is directly linked to the reduced efficiency of ventricular contractility. The latter occurs early on and is itself potentially able to perpetuate myocardial injury and loss of cardiomyocytes. OM could block this negative feedback cycle by acting directly on the systolic function, thus preventing cardiac remodelling and activation of the neurohormonal system [30,31,32]. Importantly, no relevant adverse effects regarding increase in heart rate, blood pressure or myocardial oxygen consumption have been highlighted [30,31,32] (Figure 4). 

The dose and the effects of OM administration were studied in various trials, such as the COSMIC-HF trial [33], the GALACTIC-HF trial [34] and the METEORIC-HF trial [35].

The GALACTIC-HF trial [34] aimed to examine the effects of OM administration in terms of improving symptoms, preventing clinical HF events and delaying cardiovascular death. The primary composite endpoint of hospitalization or urgent visit for HF and death from cardiovascular causes appeared to be reduced in the intervention group when compared to placebo [34]. A post-hoc analysis of the GALACTIC-HF trial was made to assess the efficacy and safety of OM in patients with severe HF [36]. In these patients, cardio-renal syndrome is common, limiting the use of the four-pillar-based HF management [36]. OM does not have an impact on kidney function or K^+^ homeostasis, thus facilitating its administration in these patients [36]. The METEORIC-HF trial [35], involving patients with HFrEF, aimed at examining their exercise capacity after OM administration, described as the change in maximal oxygen consumption (VO2) peak. The latter represents the most precise parameter to analyse the exercise capacity in patients with HFrEF. OM did not show an augmentation of exercise capacity [35]. This may be explained by the association of HF symptoms both with cardiac and extracardiac conditions, thus limiting the effect of an enhanced contractile activity on the patients’ clinical profile [35].

Sotagliflozin is a combined SGLT1i and SGLT2i, with predominantly SGLT2 inhibitory capacity [37,38]. SGLT1 mediates the reabsorption of glucose in the gastrointestinal tract, resulting in a reduction in glycaemia, insulin and glucose-dependent insulinotropic polypeptide (GIP), and an increase in plasma glucagon-like peptide 1 (GLP-1) [37,38,39,40]. The last two effects have been recently associated with an improvement in type 2 diabetes mellitus (T2DM) and cardiovascular outcomes. SGLT2 is expressed in the first proximal convoluted tubule segment, reabsorbing about 90% of the glucose in the peritubular capillaries [39]. The dual inhibition of SGLT1 and SGLT2 may provide a combined effect, causing the decrease in serum glucose levels through reduced intestinal absorption and increased urinary output [37,38,39,40]. Moreover, SGLTi possesses a myocardial anti-remodelling effect, reduces inflammation and oxidative stress and induces a cardiomyocytes metabolic shift [39,40]. Given the great impact of SGLT2i on HF treatment, the effects of Sotagliflozin were studied in the SOLOIST-WHF trial [38]. The patients enrolled in the trial were affected by T2DM and had experienced a recent worsening in HF, including both patients with HFrEF and with HF with preserved ejection fraction (HFpEF). In patients treated with Sotagliflozin, the primary endpoint, a combination of the total number of cardiovascular deaths, hospitalizations and urgent visits for HF, resulted in being significantly lower, and the benefits of an early initiation, before or immediately after hospital discharge, was highlighted [38]. The SCORED trial [41] aimed to evaluate the role of Sotagliflozin in preventing cardiovascular adverse events in patients with diabetes mellitus and chronic kidney disease. Bhatt et al. [41] demonstrated that Sotagliflozin reduced the composite endpoint of HF hospitalization, urgent ambulatory visit and cardiovascular death, along with the major incidence of adverse events such as ketoacidosis, infections and volume depletion, compared to placebo. 

Differently from Sotagliflozin, Dapagliflozin and Empagliflozin act by inhibiting selectively the function of SGLT2. These drugs have proven to have a great impact in the prognosis of HF patients, being one of the four pillars in the treatment of HFrEF [1,2] and being indicated in the treatment of HFmrEF and HFpEF, as stressed by the 2023 update of the ESC Guidelines [14]. However, to our knowledge, in the current literature the use of Empagliflozin and Dapagliflozin in the therapy of WHF in not still analysed. Two of the main trials concerning the use of these drugs, the DAPA-HF [42] and EMPEROR-Reduced [43] trials, analyse the use of Dapagliflozin and Empagliflozin in terms of time to first occurrence of hospitalization for WHF rather than in terms of the therapy of WHF itself. Moreover, the patients enrolled in these trials differ from the SOLOIST-WHF patients for their characteristics at baseline (i.e., age, LVEF, estimated glomerular filtration rate, NYHA classification and diabetes), thus making difficult the comparison among the three trials and, consequently, the analysis of the benefit in this category of HF patients [44].

A summary of the drugs studied for WHF with trials and their main findings is represented in Table 1.

## 4. The Management of Congestion: The Use of Diuretics and Some Innovative Combinations

Persistent congestion is a frequent clinical problem in patients with WHF and is related to early rehospitalization and urgent ambulatory visits [45]. The achievement of a balance in fluid status is of the essence to guarantee long-term patient stability in HF patients [45]. Moreover, patients hospitalized due to acute HF and fluid overload may have an insufficient response to loop diuretics [45]. Therefore, the possibility of optimizing the diuretic response in the early phases of acute HF hospitalization has been investigated [45,46,47,48,49,50,51,52,53,54,55,56,57,58]. On the contrary, excessive diuretic administration may be related to several complications, such as acute kidney injury and hypotension [45]. 

A growing concern in the clinical landscape is the emergence of diuretic resistance, a phenomenon characterized by the diminished effectiveness of diuretic medications over time. An insufficient diuretic response serves as a predictor for subsequent mortality, readmission or renal complications arising from HF [46]. The pathophysiological mechanisms contributing to diuretic resistance encompass an inadequately elevated daily sodium intake surpassing the salt loss induced by diuretics, hyponatremia or hypokalemic metabolic alkalosis, and the reflex activation of renal nerves [47]. Moreover, acidosis and hypoalbuminemia may impact drug delivery, whereas decreased dietary salt intake and repeated administration of furosemide reduce the renal tubular response to furosemide or affect its pharmacodynamics [48]. 

Loop diuretics are the fundamental therapy in the management of congestion. They reduce NaCl reabsorption at the level of the ascending limb of the loop of Henle by inhibiting the Na^+^-K^+^-2Cl^−^ channel in its basal membrane. The DOSE trial [49] suggested treating congestion in hospitalized patients with loop diuretics at an intravenous daily starting dose of 2.5 times the chronic oral daily dose. Despite not meeting its primary endpoints, the DOSE trial did reveal benefits in secondary endpoints, with improvements in terms of dyspnoea, congestion and NT-proBNP. In the TRANSFORM-HF trial [50], patients hospitalized due to HF and treated with either furosemide or torsemide did not show significant differences in terms of mortality or hospitalization. Hence, this suggests that appropriate loop diuretic dose may be a more important issue rather than the particular molecule to be used for treatment.

Furthermore, the addition of SGLT2i to loop diuretic therapy has been investigated and is still ongoing [51]. SGLT2i promotes osmotic diuresis by acting on the proximal tubule and mainly acts on interstitial fluid reductions, rather than depletion of intravascular fluid, which is instead the main target of loop diuretics [51]. As suggested by the EMPAG-HF [52], the EMPA-RESPONSE-AHF [53] and the EMPULSE trials [54], SGLT2i should be considered as an add-on therapy to loop diuretics in order to improve diuretic efficacy and, therefore, further facilitate decongestion in patients affected by WHF. Empagliflozin was shown to lead to a 25% increase in cumulative urine output, greater reduction in NT-proBNP and lower body weight when combined with diuretic therapy, without increased renal derangement [52,53,54]. Also, the use of Dapagliflozin in the DAPA-RESPONSE-AHF trial was associated with improvement in urinary output and symptoms in patients with acute HF [55]. 

Acetazolamide can also be considered as an add-on therapy to diuretics. Acetazolamide is a carbonic anhydrase inhibitor, which acts by reducing proximal tubular sodium reabsorption. The ADVOR trial [56] demonstrated the beneficial effects of decongesting patients with acute HF that resulted from the combination of intravenous acetazolamide and loop diuretics. The trial, however, did not explore the possibility of using intravenous acetazolamide combined with SGLT2i; hence, it is yet to be investigated whether such a combination could be beneficial [56].

Trullàs et al. [57,58] evaluated if the addition of hydrochlorothiazide (HCTZ) on top of intravenous furosemide may reduce congestion in patients admitted due to HF. The mechanism of action of HCTZ is represented by the direct inhibition of NaCl cotransporters expressed by the apical membrane on distal renal convoluted tubules. This mechanism determines the storage of Na^+^ and urine inside the tubule, promoting natriuresis and diuresis. The pharmacological effects of HCTZ start 2 h after administration, persisting up to 12 h. The effect of HCTZ is strictly dependent on renal function. The CLOROTIC trial peculiarity was to explore several endpoints related to congestion. The group who added HCTZ on top of furosemide showed significant weight loss and better 24-h urinary output, but no difference in terms of dyspnoea compared to patients treated with furosemide and placebo. Regarding the safety endpoints, patients treated with HCTZ showed increased renal function impairment, without electrolytes imbalance. No differences in terms of hospitalization and mortality were observed between the two groups [57,58].

Finerenone is a non-steroidal antagonist of mineralcorticoid receptors which hampers renin-angiotensin-aldosterone system (RAAS) activation, reducing the risk of myocardial and kidney fibrosis as well as inflammation. Compared to other MRAs it is more selective for its receptor and has a better safety profile, determining less hyperkalaemia, renal function depression and gynecomastia. Finerenone is currently indicated in patients with diabetes mellitus and chronic kidney disease, as investigated by the FIDELIO-DKD trial [59], which demonstrated that Finerenone reduced the risk of kidney disease progression and cardiovascular events, including HF hospitalization, in patients with diabetes mellitus and chronic kidney disease [59,60]. The FIGARO-DKD trial showed that Finerenone reduced the incidence of new HF and improved HF outcomes in patients with diabetes mellitus and chronic kidney disease, regardless of HF presence [61]. ARTS-HF is a IIb phase clinical trial that demonstrated that Finerenone was well tolerated and determined a significant reduction in NT proBNP levels in patients with WHF and diabetes mellitus and/or chronic kidney disease [62]. 

A summary of the main trials regarding diuretics studied for the treatment of decongestion in patients with decompensated HF is represented in Table 2.

## 5. Advances in Remote Monitoring Technologies for Heart Failure Management: Insights from CardioMEMS and Other Emerging Devices

CardioMEMS is an innovative and safe technology that consists of an implantable device placed in the pulmonary artery of the left lower lobe [63]. It measures pulmonary artery pressure (PAP) and wirelessly transmits these data to a healthcare provider. This real-time monitoring allows prompt adjustments in medication and treatment plans, mostly concerning changes in diuretic dose, in order to prevent HF exacerbations and hospitalizations; its role in HF management was stated in the latest HF guidelines with a class IIb, level B recommendation [1]. Patients with high PAP are more susceptible to decompensation, and different studies have explored the role of home telemonitoring (HTM) in this scenario [63,64,65,66].

The CHAMPION Trial [67] was a large trial which included 550 patients with chronic HF in NYHA class III with a hospital admission for HF in the previous 12 months. They were randomized, 270 patients were managed with the CardioMEMS system while 280 patients were assigned to the control group, and they were studied for at least 6 months. In the CardioMEMS group, physicians used daily measurements of PAP—in addition to standard care—in order to normalize such pressures. Instead, in the control group, no information on PAP was provided to physicians. The primary efficacy endpoint was the rate of HF hospitalization at 6 months. The primary safety endpoint was freedom from device- or system-related complications and freedom from sensor failures at 6 months. Most of these patients (73%) were male, and the mean age was 61 years. Most suffered from HFrEF, 78% had a LVEF < 40%. Relating to the baseline treatment, 90% of patients were receiving beta blockers, 78% a RAAS-inhibitor and 42% an MRAs. The trial [67] demonstrated a significant reduction in HF hospital admissions in patients with daily PAP monitoring; during the 18-months follow-up, patients treated with the usual care had a significantly higher rate of hospitalization compared to the CardioMEMS group. CardioMEMS was found to be successful from a safety point of view as well, showing freedom from device- or system-related complications equal to 98.6% and freedom from sensor failures of 100%. Furthermore, during the open-access period of the study, in which the therapy of both groups of patients was guided by the remote measurement of PAP, there was a significant reduction in hospitalization in the previously untreated group. The same promising results were achieved by the CardioMEMS Post-Approval Study (PAS) [68]. In patients treated with both GDMT and CardioMEMS, the hospitalization was significantly lower. Interestingly, these results were obtained in HF patients regardless of their LVEF, underlining the role of CardioMEMS indirectly estimating the filling pressures and volume status across the different phenotypes of HF [68]. Additionally, the MONITOR HF trial [69] was a prospective multicentre trial which enrolled patients with NYHA class III chronic HF with a previous hospital admission for decompensated HF or urgent visit requiring intravenous diuretics in the past 12 months, irrespective of LVEF. To be eligible, patients needed to be treated with OMT or maximally tolerated treatment and evaluated for an implantable cardioverter defibrillator (ICD) or cardiac resynchronization therapy (CRT), if indicated. In the trial, 348 patients were randomized in a 1:1 fashion, with 179 being assigned to the CardioMEMS group and 178 being assigned to the control group (being treated with GDMT and diuretics). The primary endpoint was the mean change in KCCQ-12 scores from baseline to 12 months. The MONITOR-HF trial was carried out later on compared to the CHAMPION trial, hence in an era in which the use of the quadruple HF therapy was much more established. Therefore, the MONITOR-HF trial demonstrated the added value that CardioMEMS displays even in a setting of OMT. The trial [69] has shown that CardioMEMS improved the QoL of HF patients, with a significant increase in KCCQ-12 scores when compared to controls. Furthermore, frequent measurements of PAP may be crucial to understand the real impact of GDMT in the pulmonary hemodynamic. The EMBRACE-HF trial [70] was a multicentre trial whose primary endpoint was change in pulmonary artery diastolic pressure (PADP) from baseline to the end of treatment in patients with HF and previously implanted CardioMEMS being treated with either Empagliflozin or placebo. In the trial, 65 patients were randomized, with 33 being treated with Empagliflozin and 32 with placebo. The trial [70] showed a prompt reduction in pulmonary pressures in patients treated with Empagliflozin, strengthening their decongestive power. 

In addition to CardioMEMS, new devices have proven to be capable of collecting physiological data, offering novel perspectives in the treatment of HF.

For instance, it is well known that high filling pressures in the left atrium (LA) contribute to congestion and the worsening of HF symptoms, mainly in patients with HFpEF, making it reasonable to monitor its changes through LA pressure sensors (Heart POD and V-LAP devices). Intracardiac pressure measurements could offer a more nuanced evaluation of critical clinical events in patients with HF. This includes assessing diastolic function, identifying atrial arrhythmias, severity and the deterioration of mitral regurgitation. The V-LAP system incorporates a sensory implant that wirelessly measures and transmits left atrial pressure (LAP) to an external unit. The VECTOR-HF Study [71] showed that V-LAP safely provided reliable measurements in patients with NYHA Class III; the patients enrolled in the study, furthermore, underwent right heart catheterization and a significant correlation was found between the mean LAP and mean pulmonary capillary wedge pressure (PCWP). The data obtained by the device may allow the remote tailoring of the therapy, and preliminary data showed promising results in increasing NYHA functional class status and QoL [72].

Moreover, currently under study is an implantable sensor placed in the inferior vena cava (IVC) in order to constantly monitor fluid accumulation and create a new way to detect intravascular volume expansion. In particular, the FIRE1 system consists of an embedded sensor and an external electronic system that utilize a radiofrequency antenna integrated into an external belt. The recorded data are processed and stored into a cloud, providing real-time information on the IVC area. However, the study was conducted on animals, and data from studies on humans are not yet available [73].

## 6. When Pharmacological Therapy Is Not Enough: Cardiac Contractility Modulation and Baroceptor Activation Therapy

In recent years, a new implantable device has shown promising effects in the HF setting. Cardiac contractility modulation (CCM) represents a device-based therapy suitable for a wide range of HF patients that do not meet the criteria for CRT [74]. This device is reserved for those with LVEF between 25% and 45% who remain symptomatic (NYHA II–IV) despite OMT and who do not have a left bundle branch block (LBBB) morphology [1,2,75]. The CCM device physically resembles a pacemaker, as it is constituted by a generator and two leads; the generator, containing the battery and electronic circuits, similar to any other cardiac implantable electronic device (CIED) generator, can be positioned in the left or right pectoral region, in a pocket located under the skin. Two ventricular active fixation leads complete the CCM structure: during the implantation procedure these two leads are inserted through the subclavian vein and must be screwed into the right ventricular septum. 

However, the CCM function differs from pacemakers as it generates an electrical signal specifically and exclusively during the absolute refractory period of the myocardium for 5 to 7 h a day. It does not capture the myocardium in order to obtain heart contractions, because impulses are delivered during the non-excitatory period, but acts at the intracellular level. Chronic HFrEF induces a phenotypic transition in cardiomyocytes towards a foetal pattern, with a subsequent enhanced expression of BNP and reduced levels of Protein Kinase A (PKA) [76]. Low levels of PKA determine low phosphorylation of phospholamban with inhibition of sarco/endoplasmic reticulum Ca^2+^ATPase (SERCA2a) channels. Chronic HF also is characterized by low synthesis of SERCA2A and ryanodine receptor 2 channels (RYR2). The common final effect is the reduced availability of intracellular Ca^2+^ with a resulting reduction in myocardial contractility [76].

CCM manages to enhance Ca^2+^ handling through the upregulation of RYR2 and SERCA2a, restoring the function of the Na^+^/Ca^2+^ exchanger and normalizing the levels of phospholamban phosphorylation [77]. These different actions enhance cellular Ca^2+^ availability and improve myocardial contractility without increasing myocardial oxygen consumption, but they do not directly influence the LVEF [78,79]. Instead of pacemakers, CCM can be compared to Levosimendan for its effect on the enhancement of myofilament calcium sensitivity [80]. 

CCM has different time-dependent effects: in the first minutes to hours after delivery of electrical signals it causes an increase in phosphorylation of phospholamban with increased contractility in the local myocardium. Weeks after CCM implantation, a genetic remodelling is observed in myocardial regions further away from the CCM electrodes. Within weeks to months, CCM generates a global myocardial reverse remodelling mixing its inotropic and genotropic effects [76,78,79].

While CCM improves functional exercise capacity and exercise tolerance, it is mainly recognized for its effects on the reduction in the number of HF hospitalizations [81]. 

The trials with the most encouraging results are the FIX-HF-4 study [82] and the FIX-HF-5 trial [81], which examined CCM as an addition to OMT compared to OMT alone, demonstrating that CCM is safe and effective in improving exercise tolerance and QoL, while reducing HF hospitalizations. More specifically, the FIX-HF-4 study [82] was a multicentre trial where 164 subjects were included; 80 were randomized to group 1 (with CCM on for the first 12 weeks) and 84 were randomized to group 2 (with CCM off in the first 12 weeks). Patients were considered eligible if they were >18 years of age and had symptomatic HF (NYHA class ≥ II), ischemic or idiopathic cardiomyopathy, LVEF ≤ 35% and peak oxygen uptake between 10 and 20 mL O_2_/min/kg. They were also required to be undergoing stable HF medical treatment. In terms of adverse events, the most common ones were episodes of decompensated HF, atrial fibrillation, bleeding at the CCM implant site and pneumonia. The number or types of adverse events were not significantly different between the two groups. The FIX-HF-5 study [81] was a prospective, randomized trial of OMT alone as compared with OMT plus CCM in outpatients with medically refractory HF in NYHA class III or IV, with a LVEF between 25% and 45%, and with sinus rhythm and a QRS < 130 msec. Also in this case, no significant differences were reported in terms of adverse events occurrence. In both studies, patients were followed up for 24 weeks. These two studies demonstrated that CCM is safe and effective in improving exercise tolerance and QoL, while reducing HF hospitalizations. However, these studies do have certain limitations. First of all, the limited follow-up period provides a limited possibility of evaluating the long-terms effects that CCM might display on mortality and hospitalizations. Secondly, the significant amount of medical attention by the treating personnel might have contributed to a placebo effect. Moreover, being so closely observed and followed-up might have also had a significant effect on lowering the rate of hospitalizations and mortality. Moreover, in the FIX-HF-4 study, the mean age of patients was 59 years, and they were predominantly males. This might have underrepresented the real-life HF population, as it includes older individuals and a more significant proportion of females. Even if CCM provided beneficial effects in terms of functional capacity and QoL [83], more randomized controlled trials are needed to support their systematic use. CCM has also shown promising effects in complex settings such as HF in cardiac amyloidosis [84] or HF in patients following heart transplant [85]. 

HF is characterized by an imbalance between the sympathetic and parasympathetic activity, with an increased function of the former. Baroreflex activation therapy (BAT) consists of a device implanted to electrically stimulate the baroreceptors responsible for autonomic nervous system modulation. The device typically includes a lead or electrode that is placed near the baroreceptor region and connected to a pulse generator, which is implanted under the skin. The pulse generator delivers controlled electrical impulses to the baroreceptor area, mimicking the signals that the body’s natural baroreflex would produce. The electrical stimulation of the baroreceptors leads to various physiological effects, including a reduction in sympathetic nervous system activity (which tends to increase heart rate and blood pressure) and an increase in parasympathetic activity (which tends to decrease heart rate and promote relaxation). As a result, blood pressure is lowered, and the overall cardiovascular system experiences a more balanced regulation [86]. The Barostim Neo™ device has shown promising results in safely improving clinical symptoms and NYHA functional class in patients with worsening HF [87]. The BeAT-HF study [88] and the HOPE4HF study [89], for instance, have revealed that BAT therapy significantly improved QoL and exercise capacity, and reduced NT-proBNP levels. 

A summary of the main trials regarding invasive monitoring systems and device therapy used in WHF is represented in Table 3. 

## 7. Conclusions

**Figure 5:**
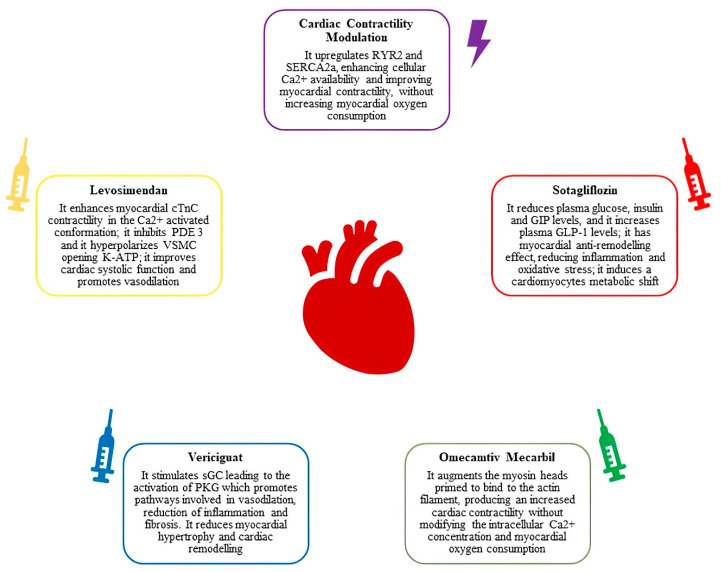
Molecular and pathophysiological features of pharmacological agents and devices for WHF. Repetitive Levosimendan infusion, Sotagliflozin, Vericiguat and Omecamtiv Mecarbil are pharmacological agents tested in trials involving WHF patients. CCM is a device used to treat patients with WHF symptoms and signs despite background OMT. WHF: worsening heart failure; CCM: cardiac contractility modulation; OMT: optimized medical therapy; RYR2: Ryanodine receptor 2; SERCA2a: sarco/endoplasmic reticulum Ca^2+^ATPase; cTnC: cardiac troponin C; PDE 3: phosphodiesterase 3; VSMC: vascular smooth muscle cell; K-ATP: ATP-sensitive potassium channels; sGC: Soluble guanylyl cyclase; PKG: protein kinase G; GIP: Gastric inhibitory polypeptide; GLP-1: glucagon-like peptide-1.

HF has a complex pathophysiology [90], and several molecular mechanisms, which are only partially known, are involved in this syndrome. Neurohormonal, RAAS and adrenergic system hyperreactivity are well known pathways involved in HF. Several drugs have been developed to inhibit these pathways, drastically improving patients’ life expectancy. The first-line strategy in HF therapy involves a comprehensive approach that integrates key medications (BB, MRAs, ARNI and SGLT2i) to address the multifaceted aspects of the condition and has been shown to significantly reduce mortality and hospitalization [1]; when feasible, all the four cornerstone drugs should be started as soon as possible and up-titrated to the maximum tolerated dose [91]. These drugs have been shown to display beneficial effects early on, even at low starting dosages, hence underlining the importance of promptly starting an OMT as soon as possible [14]. Despite these advances, a non-neglectable subset of patients with HF are exposed to a residual risk of WHF [15]. This aspect has provided an onrush to investigate novel molecular mechanisms and therapeutic targets. The introduction of stimulator drugs, such as Vericiguat and OM, are interesting new tools to treat HF in the near future and are particularly promising for certain HF subgroups. Another relevant support for OMT is provided by devices such as CardioMEMS and CCM, which have been shown to ameliorate patients’ QoL and to reduce the rate of hospitalizations and mortality. These devices are of particular relevance considering they can be offered to patients who do not fit the criteria for CRT but who, at the same time, are subject to some residual risk despite OMT. An additional important aspect in patients with WHF is residual congestion treatment, and new diuretics, as well as changes in the diuretic therapy approach, may improve this issue. HF constitutes a substantial burden on healthcare systems, and its impact varies across different healthcare frameworks; the burden of HF underscores the importance of resource allocation efficiency and the need for equitable access to treatments. As is already well-known, a relevant issue for patients with worsening HF is the frequent rate of hospitalization due to acute decompensations. The use of repetitive cycles of Levosimendan as a background therapy, on top of which adding OMT, has been suggested as an approach aimed at overcoming the high burden of such events. The management of WHF is at a critical juncture, and it is evident that a multifaceted approach is necessary, one that combines established strategies with novel interventions (Figure 5), so as to work synergically to target all the molecular pathways known to play a role in this complex syndrome. These new therapeutic measures, whether in the form of advanced pharmacological agents, device-based interventions or innovative monitoring technologies, are paving the way for a more personalized and effective approach to patient care.

## Figures and Tables

**Table 1:** Summary of drugs studied in WHF with trials and their main findings.

Drug	Trials and Main Findings	Reference
**Levosimendan**	**LevoRep trial**: Intermittent Levosimendan infusion did not significantly improve patients’ functional capacity and quality of life.	[20]
	**LION-HEART trial**: Intermittent Levosimendan administration was effective in decreasing the serum concentration of NT-proBNP and was associated with improvement in clinical symptoms.	[21]
	**LAICA study**: Repetitive Levosimendan infusion is effective in reducing the HF hospitalization rate and HF worsening in patients with advanced HF.	[22]
	**LeoDOR trial**: Repetitive Levosimendan administration in patients with a recent HF hospitalization did not result in post-hospitalization clinical stability.	[23]
**Vericiguat**	**VICTORIA trial**: The primary composite of death from cardiovascular causes or first hospitalization for HF was lower in those who received Vericiguat.	[27]
**Omecamtiv Mecarbil**	**GALACTIC-HF trial**: The primary composite endpoint of hospitalization or urgent visit for HF and death from cardiovascular causes is reduced in patients treated with OM.	[34]
	**METEORIC-HF trial**: OM did not increase exercise capacity after 20 weeks of treatment.	[35]
	**COSMIC-HF trial**: Guiding the administration of OM through drug pharmacokinetic is associated with reduction in ventricular diameter and cardiac function improvement.	[33]
**Sotagliflozin**	**SOLOIST-WHF trial**: The primary endpoint, a combination of total number of cardiovascular deaths, hospitalizations and urgent visits for HF, resulted lower, and the benefits of an early initiation of Sotagliflozin, before or immediately after hospital discharge, was highlighted.	[38]
	**SCORED trial**: Sotagliflozin reduced the composite endpoint of HF hospitalization, urgent ambulatory visit and cardiovascular death, along with major incidence of adverse events.	[41]

WHF: worsening heart failure; NT proBNP: N-terminal pro B-type natriuretic peptide; HF: heart failure; OM: omecamtiv mecarbil.

**Table 2:** Summary of drugs with diuretic properties studied in WHF with trials and their main findings.

Drug	Trials and Main Findings	Reference
**SGLT2i**	**EMPAG-HF**: In patients with acute decompensated HF, the early initiation of Empagliflozin on top of standard diuretic improves urinary output.	[52]
	**EMPA-RESPONSE-AHF**: In acute HF patients, the use of Empagliflozin is associated with reduction in WHF, death and rehospitalization, and increased urinary output.	[53]
	**EMPULSE trial**: Starting Empagliflozin in patients admitted for AHF is associated with early and effective decongestion.	[54]
	**DAPA-RESPONSE-AHF**: In patients with acute HF, Dapagliflozin is associated with diuresis and symptoms improvement.	[55]
**Acetazolamide**	**ADVOR trial**: In patients with acute decompensated HF, the addition of Acetazolamide on top of furosemide is associated with increased rate of decongestion.	[56]
**HCTZ**	**CLOROTIC trial**: Patients treated with HCTZ on top of intravenous furosemide show significant weight loss and better 24-h urinary output, but no difference in terms of dyspnoea.	[57,58]
**Finerenone**	**FIDELIO-DKD**: Finerenone reduces the risk of kidney disease progression and cardiovascular events, including HF hospitalization, in patients with diabetes mellitus and chronic kidney disease.	[59]
	**FIGARO-DKD**: Finerenone reduces the incidence of new HF and improved HF outcomes in patients with diabetes mellitus and chronic kidney disease, regardless of HF presence.	[61]
	**ARTS-HF**: Finerenone determines a significant reduction in NT proBNP levels in patients with WHF and diabetes mellitus and/or chronic kidney disease.	[62]

WHF: worsening heart failure; HF: heart failure; AHF: acute heart failure; WHF: worsening heart failure; HCTZ: Hydrochlothiazide; NT proBNP: N-terminal pro B-type natriuretic peptide; SGLT2i: sodium glucose cotransporter 2 inhibitors.

**Table 3:** Summary of invasive monitoring systems and device therapy used in WHF. For these systems, the main trials and their main findings are described.

Device	Trials and Main Findings	Reference
**CardioMEMS**	**CHAMPION trial**: Significant reduction in HF hospital admissions in patients with daily PAP monitoring through CardioMEMS was observed.	[67]
	**MONITOR-HF trial**: CardioMEMS improves the QoL of HF patients with a significant increase in KCCQ-12 score.	[69]
	**EMBRACE-HF trial**: A prompt reduction in PAP in patients treated with Empagliflozin and monitored with CardioMEMS was observed.	[70]
**V-LAP**	**VECTOR-HF study**: A significant correlation was found between the mean LA pressure and mean PCWP.	[71]
**CCM**	**FIX-HF-4 study and FIX-HF-5**: CCM is safe and effective in improving exercise tolerance and QoL, while reducing HF hospitalizations.	[81,82]
**BAT**	**BeAT-HF study and HOPE4HF study**: BAT therapy significantly improves QoL, exercise capacity and reduced NT-proBNP levels.	[88,89]

HF: heart failure; PAP: pulmonary artery pressure; LA: left atrial; KCCQ-12: Kansas City cardiomyopathy questionnaire; PCWP: pulmonary capillary wedge pressure; QoL: quality of life; NT pro BNP: N-terminal pro B-type natriuretic peptide; CCM: cardiac contractility modulation; BAT: baroreflex activation therapy.
